# Supplementary material for: Effects of a Midwife-Coordinated Maternity Care Intervention (ChroPreg) vs. Standard Care in Pregnant Women with Chronic Medical Conditions: Results from a Randomized Controlled Trial
Source: Int J Environ Res Public Health. 2021 Jul 25;18(15):7875. doi: 10.3390/ijerph18157875 (PMC8345548; doi:10.3390/ijerph18157875)
Supplement: Supplementary file 1 [file ijerph-18-07875-s001.zip › ijerph-1265941-supplementary.pdf]

Supplementary Materials

Table S1. Consultations/activities in the ChroPreg group and Standard Care group delivered by midwives.

	**ChroPreg group**<br>Focus points of consultation	**Standard Care group**<br>Focus points of consultation
Week 14-18<br>*Face-to-face*<br>30 minutes	Medical history taking, follow up on information from the general practitioner<br>Care-coordination	Medical history taking, follow up on information from the general practitioner
Week 20–24<br>*Face-to-face*<br>1 hour	Psychological well-being (experience of pregnancy, prenatal attachment)<br>preparation for breastfeeding (including discussion of medication).<br>Interaction between pregnancy and chronic disease, usual pregnancy symptoms.<br>Care-coordination	None
Week 28-30<br>*Face-to-face **<br>30 minutes	Breastfeeding, rhesus prophylaxis, well-being, and symptoms of pregnancy and chronic disease.<br>Care-coordination	Breastfeeding, rhesus prophylaxis, well-being, and symptoms of pregnancy
Week 30–33<br>*Face-to-face*<br>1 hour	Individual birth plan<br>Care coordination	None
Week 35-36<br>30 minutes	Pregnancy development, well-being, symptoms of CMC, and the coming birth<br>Care-coordination.	Pregnancy and the coming birth, and well-being.
Week 38-39<br>*Face-to-face*<br>30 minutes	Pregnancy development, well-being, symptoms of CMC, and the coming birth<br>Care-coordination.	Pregnancy and the coming birth, and well-being.
Week 40<br>*Face-to-face*<br>30 minutes	Pregnancy development, well-being, symptoms of CMC, and the coming birth<br>Care-coordination.	Pregnancy and the coming birth, and well-being.
1st and 2nd week after discharge from hospital<br>Telephone	Breastfeeding, CMC management, medication, and psychological well-being.<br>Care-coordination	The woman can contact the hospital with questions and need for a postpartum check-up.
Postpartum debriefing session<br>*Face-to-face/telephone*<br>1 hour	This visit is arranged for the woman to have the opportunity to discuss her experiences of pregnancy and childbirth. The midwife will have medical records available, but the focus is to allow the woman to talk and elaborate on her experience. If the midwife and the woman identify a need for further counseling, the referral can be made to a psychologist.	No routine follow-up.<br><br>The woman can contact the Department for an appointment to discuss experiences of pregnancy and childbirth.
E-mail correspondence<br>Unlimited	The woman can contact the specialized midwife by e-mail. This contact may concern all relevant issues during pregnancy and postpartum.	No e-mail correspondence included.<br>Women can contact the coordinating midwife with questions and concerns
Weekly telephone hours<br><br>Unlimited	One day weekly, the midwife is available for telephone consultation Participants can call the midwife with questions or concerns. The midwife will evaluate if further visits will be needed in addition to the telephone contacts.	Women can contact the coordinating midwife with questions and concerns.

*All standard midwife appointments from week 28 and on also include urine testing for glucose and protein and measuring blood pressure (screening for indicators for gestational diabetes and pre-eclampsia) and physical examinations (measuring the size, heartbeat, and position of the fetus).
